# COL5A2 is a prognostic-related biomarker and correlated with immune infiltrates in gastric cancer based on transcriptomics and single-cell RNA sequencing

**DOI:** 10.1186/s12920-023-01659-9

**Published:** 2023-09-18

**Authors:** Meiru Chen, Xinying Zhu, Lixian Zhang, Dongqiang Zhao

**Affiliations:** 1 Department of Gastroenterology, The Second Hospital of Hebei Medical University, Shijiazhuang, Hebei Province 050000 China; 2 Department of Gastroenterology, Hengshui People’s Hospital, Hengshui, Hebei Province 053000 China; 3 Department of Gastroenterology, The Third Hospital of Hebei Medical University, Shijiazhuang, Hebei Province 050000 China

**Keywords:** COL5A2, Gastric cancer (GC), Prognosis, Immune cell infiltration, Immunotherapy

## Abstract

**Background:**

There is still a therapeutic challenge in treating gastric cancer (GC) due to its high incidence and poor prognosis. Collagen type V alpha 2 (COL5A2) is increased in various cancers, yet it remains unclear how it contributes to the prognosis and immunity of GC.

**Methods:**

The Cancer Genome Atlas (TCGA) and Gene Expression Omnibus (GEO) datasets were used to download transcriptome profiling (TCGA-STAD; GSE84437), single-cell RNA sequencing (scRNA-seq) data (GSE167297) and clinical information. COL5A2 expression and its relationship with clinicopathological factors were analyzed. We conducted survival analysis and Cox regression analysis to evaluate the prognosis and independent factors of GC. Co-expressed analysis was also performed. To identify the underlying mechanism, we conducted analyses of differentially expressed genes (DEGs) and functional enrichment. The correlations between COL5A2 expression and immune cell infiltration levels and immune infiltrate gene marker sets were further explored. Additionally, we analyzed the association of COL5A2 expression with immunological checkpoint molecules. Furthermore, the relationship between COL5A2 expression and immunotherapy sensitivity was also investigated.

**Results:**

COL5A2 expression was elevated in GC. More than this, the scRNA-seq analysis revealed that COL5A2 expression had a spatial gradient. The upregulated COL5A2 was associated with worse overall survival. A significant correlation was found between COL5A2 overexpression and age, T classification and clinical stage in GC. COL5A2 was found to be an independent factor for the unfortunate outcome in Cox regression analysis. The co-expressed genes of COL5A2 were associated with tumor stage or poor survival. Enrichment analysis revealed that the DEGs were mainly associated with extracellular matrix (ECM)-related processes, PI3K-AKT signaling pathway, and focal adhesion. GSEA analyses revealed that COL5A2 was associated with tumor progression-related pathways. Meanwhile, COL5A2 expression was correlated with tumor-infiltrating immune cells. Moreover, immunophenoscore (IPS) analysis and PRJEB25780 cohorts showed that patients with low COL5A2 expression were highly sensitive to immunotherapy.

**Conclusions:**

COL5A2 might act as a prognostic biomarker of GC prognosis and immune infiltration and may provide a therapeutic intervention strategy.

**Supplementary Information:**

The online version contains supplementary material available at 10.1186/s12920-023-01659-9.

## Introduction

GC is one of the most common malignancies that has a poor prognosis. The incidence and mortality are fifth and fourth globally [1]. According to the 2020 global statistics, the number of new cases of GC has exceeded a million, and about 769,000 patients died from GC [1]. Despite major advances in surgery, chemical therapy, radiotherapy, and neoadjuvant therapy, patients with GC (particularly those with advanced GC) do not have a good prognosis [2–5]. Recently, along with the discovery of monoclonal antibodies and small molecule inhibitors, targeted therapy interventions have become more accessible, and have been an important therapy for patients with GC [6]. Hence, a sensitive and novel biomarker that can accurately predict the prognosis and treatment for GC is an absolute necessity.

Collagen is the most plentiful protein in human tissue and is the basal component of the extracellular matrix (ECM) architecture [7]. Researchers have determined that the ECM contributes to the initiation, progression, and migration of cancer [7]. The collagen type V alpha 2 (COL5A2) belongs to the collagen type 5 family, locates at 2q32.2. A low abundance fibrillar collagen alpha chain is encoded by this gene. This gene's mutations were linked to the Ehlers-Danlos syndrome and a genetically complex autoimmune disease [8, 9]. COL5A2 shows abnormal expression in tumors, which affects malignancy and progression [10–12]. Nevertheless, the clinical significance of COL5A2 in GC and its molecular mechanism are not well understood.

COL5A2 was tested for its prognostic value in GC in this study. The TCGA and GEO databases were used to acquire RNA-seq and microarray data. We examined COL5A2 expression in human stomach adenocarcinoma (STAD) samples. In addition, the scRNA-seq analysis was also conducted to detect the expression of COL5A2. COL5A2 expression level and clinicopathological characteristics, along with prognosis, were comprehensively analyzed. We conducted differential expression analysis based on the expression of COL5A2, along with Gene Ontology (GO) and Kyoto Encyclopedia of Genes and Genomes (KEGG) enrichment analyses of these DEGs, as well as Gene Set Enrichment Analysis (GSEA) to fully study the biological functions of COL5A2 in GC. Moreover, the further investigation suggested that COL5A2 expression was associated with tumor-infiltrating immune cells, immune marker sets and immune checkpoint molecules, and may affect the immunotherapy response. These findings may provide an underlying mechanism by which COL5A2 contributes to GC progression and immune tolerance. Hence, for patients with GC, COL5A2 can be a promising biomarker and therapeutic target.

## Materials and methods

### Data extraction from TCGA and GEO database

Transcriptome profiling and clinical data of STAD patients were downloaded from the TCGA website (https://portal.gdc.cancer.gov). The Perl (http://www.perl.org/) script was used to merge the RNA-seq data files into a matrix file. Then 375 samples of GC samples and 32 adjacent nontumor samples were obtained. A Perl script was used to convert Ensemble IDs (http://ensembl.org/index.html) into gene symbols. We searched the GEO database (https://www.ncbi.nlm.nih.gov/geo/) with the keywords “gastric cancer survival” and “homo sapiens”, and downloaded “GSE84437” which had the maximum dataset.

A set of raw scRNA-seq data, GSE167297, was downloaded from the GEO database for scRNA-seq analysis, which contained 5 deep layer samples, 5 superficial layer samples and 4 normal gastric samples from 5 patients. Seurat package (V4.1.1) in R was used to perform the quality control (QC) process. The raw count matrix was converted into a Seurat object. With the “PercentageFeatureSet” function, the percentage of mitochondrial genes, ribosomal genes and haemoglobin genes were calculated. We excluded cells expressing less than 300 genes, and genes expressed in fewer than three cells, as well as noncoding genes. Mitochondrial gene content > 15%, ribosomal gene content > 3% and haemoglobin gene content > 1% were also excluded. We normalized the filtered data using “LogNormalize” with 10,000 scales. Then, based on the top 2000 highly variable genes, an analysis of principal components (PCA) was conducted. A batch effect was corrected using the harmony R package. To identify cell types, Uniform Manifold Approximation and Projection (UMAP) and marker gene analyses were carried out [13]. The UMAP plots identified 17 cell clusters with a resolution of 0.8. We curated the cell clusters based on known lineage markers, such as T cell (CD3D, CD3E, PTPRC), B cell (MS4A1), myeloid cell (CD68, CD163, CD14, CD86, TPSAB1), plasma (MZB1, SDC1, CD79A), epithelial cell (EPCAM, KRT19, PROM1, ALDH1A1), endothelial cell (VWF) and fibroblasts (FGF7, ACTA2). The expression of COL5A2 was visualized with ggplot2 and clustree package in R. A chi-square test with rcompanion package in R was used to determine whether statistically significant differences existed.

### COL5A2 differential expression in patients with GC

Based on TCGA expression profile data, 33 cancers were evaluated for their COL5A2 mRNA levels via Tumor Immune Estimation Resource (TIMER) 2.0 (http://timer.cistrome.org/) [14]. We analyzed the expression of COL5A2 in normal and GC samples along with 27 pairs of GC and adjacent samples were conducted via limma and ggplot2 packages in R software.

### Correlation analysis of COL5A2 and clinicopathological factors

To investigate whether the mRNA expression of COL5A2 correlates with the clinicopathological factors of GC patients, we explored clinical information of 375 GC samples extracted from TCGA and verified the results with 433 GC samples using GSE84437 via limma package in R software.

### Immunohistochemistry (IHC)

A total of 30 GC and 10 normal gastric tissues as controls were selected from The Second Hospital of Hebei Medical University, Hebei Province, China, between June 2022 and June 2023. Ethics approval for the study was obtained from the institution. Pathological examinations confirmed the diagnosis of gastric cancer in all patients. No prior treatment was given to any of the patients before undergoing surgical resection. Samples from patients were prepared as formalin-fixed paraffin-embedded (FFPE) sections and the IHC staining against COL5A2 was performed as previously described [15]. Anti-COL5A2 (1:50, OriGene, #TA313657) was then incubated overnight at 4°C with the samples. After incubation with the secondary antibodies (ZSGB-BIO, PV-9000), the sections were stained with DAB (ZSGB-BIO, ZLI-9017) the following day. We evaluated the staining results as described previously [16]. There were four scores for staining intensity in each field: 0 was no staining; 1 was weakly positive; 2 was moderately positive; 3 was strongly positive. In addition, positive cells were divided into five grades (percentage scores): 0–10% (0), 11–25% (1), 26–50% (2), 51–75% (3), and > 75% (4). An overall staining score was calculated by multiplying the intensity score by the percentage score of protein staining. High COL5A2 expression was defined as a final score of ≤ 4, and low COL5A2 expression was defined as a final score of > 5. The immunostaining was evaluated by two independent pathologists who were blind to the study's findings.

### Survival and significant prognostic marker analyses of COL5A2

The samples were classified as high-expression and low-expression groups according to COL5A2 expression levels above or below the median value. In this study, based on the TCGA datasets and GSE84437, we conducted survival analyses to evaluate the impact of COL5A2 expression on GC survival. Univariate and multivariate Cox regression analyses were performed to determine independent prognostic factors. The prognostic characteristics of COL5A2 and clinical characteristics were assessed by calculating the area under the receiver operating characteristic (ROC) curve (AUC) for GC patients in TCGA-STAD. The statistical analyses were conducted using the R software. The limma, survival, survminer and timeROC packages in R software were used appropriately to conduct the statistical analyses. A *P*-value < 0.05 was considered significant.

### Analysis of COL5A2 co-expressed genes in GC

For further exploration of the COL5A2-associated molecular mechanism, COL5A2 co-expressed genes were identified with the cBioPortal database (https://www.cbioportal.org/). The top six significant COL5A2 co-expressed genes were used for further analysis, and the TIMER2.0 database was used to verify their correlation with COL5A2 expression. In addition, by using Gene Expression Profiling Interactive Analysis (GEPIA) (http://gepia.cancer-pku.cn/index.html), we explored COL5A2 co-expressed genes in GC and the relationship between these genes and tumor stages. Moreover, we analyzed the prognosis significance of the six co-expression genes via Kaplan–Meier plotter online database (http://kmplot.com/analysis/).

### Analysis of differentially expressed genes and functional enrichment

We conducted differential expression analysis with the limma R package. Statistical significance was determined based on adjusted *P*-value < 0.05 and logFC > 1 [17]. To define the biological functions of DEGs and COL5A2-related genes, GO and KEGG [18] enrichment analyses were conducted via clusterProfiler R package [19]. The package was also used to perform GSEA analysis for the investigation of COL5A2 potential regulatory mechanisms. We downloaded the “h.all.v7.4.symbols.gmt” gene sets from the GSEA website. The terms with a *p*-value < 0.05 were selected.

### Relationship between COL5A2 and tumor immunoreactivity

To investigate the correlation between COL5A2 and 28 tumor-infiltrating lymphocytes (TILs), we employed the TISIDB online platform (http://cis.hku.hk/TISIDB/) [20]. Additionally, the relationship between COL5A2 and immune cell infiltration levels, including CD4 + and CD8 + T cells, Treg cells, mast cells, dendritic cells (DCs), macrophages, monocytes, and neutrophils was conducted via the TIMER2.0 database. Moreover, the relationships between COL5A2 expression and tumor-infiltrating immune cell gene markers were analyzed through the TIMER2.0 database. The markers of CD8 + T cells, T cells (general), B cells, monocytes, tumor-associated macrophages (TAMs), M1 and M2 macrophages, neutrophils, natural killer (NK) cells, DCs and T cells (Th1, Th2, Tfh, Th17, Tregs, and exhausted T cells) with different function were contained. The immune gene marker sets were quoted in prior studies [21–23].

The Gene Expression Profiling Interactive Analysis (GEPIA) (http://gepia.cancer-pku.cn/index.html) [24] was used to further confirm the significant correlations found in TIMER2.0. We explored the gene expression correlations via Spearman’s correlation analysis with both tumor and normal tissues.

Moreover, the association between COL5A2 expression and the 47 common immune checkpoint molecules was examined via R software with limma, reshape2, ggplot2, ggpubr, and corrplot packages.

### Predict the sensitivity to immunotherapy

According to previous research, IPS can predict the efficacy of immunotherapy [25]. IPS for GC patients are available through The Cancer Immunome Atlas (TCIA) (https://tcia.at/home). The limma and ggpubr packages in R software were used for analysis. Additionally, as a part of our analysis of the predictive value of COL5A2 expression in immunotherapy response, we downloaded RNA-Seq data of the immunotherapy cohort PRJEB25780 from European Nucleotide Archive (ENA) (https://www.ebi.ac.uk/ena/browser/home). In this cohort, researchers examined 45 patients with metastatic or recurrent gastric cancer who received anti-programmed cell death protein 1 (PD-1) therapy [26]. The immunotherapy responses were classified into four types: complete response (CR), partial response (PR), stable disease (SD) and progressive disease (PD). We compared COL5A2 expression levels between the group of patients with the confirmed response (CR/PR) and those without clinical response (PD/SD) through limma and ggpubr packages in R software. Furthermore, we derived the ROC curve of COL5A2 expression to predict the response to anti-PD-1 therapy for GC patients through pROC packages in R software.

### Statistical analysis

In this study, scRNA-seq analysis was conducted with R software (version 4.1.3), and the rest statistical analyses were conducted using R software (version 4.1.2) and Perl (version 5.30.0). Throughout the study, the appropriate R packages and statistical methods were described. Statistical analyses involved Chi-square tests with SPSS version 26.0 (IBM Corp, Armonk, NY). We considered *P* < 0.05 to be statistically significant.

## Results

### Abnormally overexpression of COL5A2 in GC

**Fig. 1:**
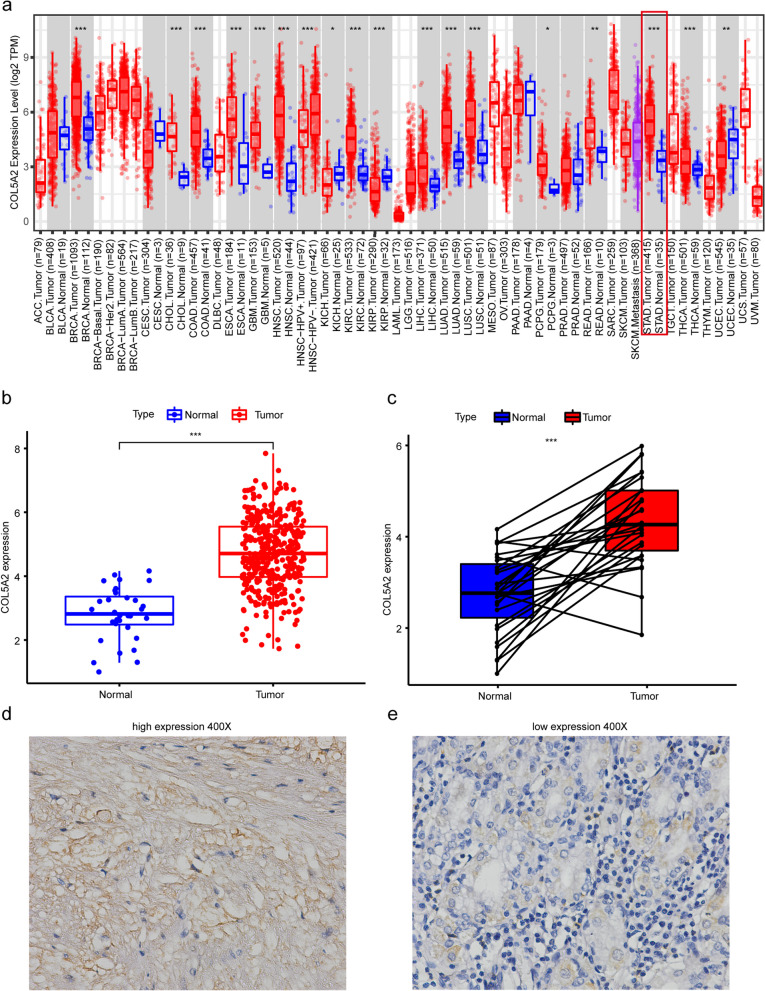
The expression level of COL5A2 was upregulated in gastric cancer (GC). **a** The level of COL5A2 expression in different tumor types analyzed in TIMER2.0. **b** COL5A2 mRNA levels in GC samples and normal gastric samples. **c** The expression of COL5A2 in GC samples was significantly higher than that of 27 pairs of non-cancerous adjacent samples (*p* < 0.001). **d** High expression of COL5A2 in GC tissues. **e** Low expression of COL5A2 in normal tissues. The magnification was 400 fold. *, *P* < 0.05; **, *P* < 0.01; ***, *P* < 0.001

Firstly, to fully evaluate the significance of COL5A2 in various solid tumors, pan-cancer analysis was conducted to compare COL5A2 mRNA expression levels between tumor samples and normal samples of the TCGA-STAD through the TIMER2.0 database. According to the analysis, COL5A2 was significantly upregulated in cancerous tissues, such as STAD, cholangiocarcinoma, thyroid cancer and colon adenocarcinoma. It suggested that COL5A2 in these cancers could be an oncogene (Fig. 1a). Secondly, the TCGA-STAD dataset allowed us to compare COL5A2 expression in 32 paracancerous and 375 GC samples. Based on the scatter plot, we found that COL5A2 expression in GC samples was significantly higher than that in normal tissues (*p* < 0.001, Fig. 1b). Following this, the data from 407 samples was then used to find 27 pairs of GC and adjacent normal samples. It was revealed that COL5A2 was significantly upregulated in GC samples compared to matched paracancerous samples (*p* < 0.001, Fig. 1c). In addition, COL5A2 protein expression was examined in GC tissues and peritumoral tissues via IHC (Fig. 1d-e). High COL5A2 expression was detected in GC tissues (42.5%, 17/30), but not detected in adjacent non-tumor tissues (0%, 0/10). Chi-square tests and *p* values were calculated (chi-square = 4.685, *P* = 0.03). Overall, the findings revealed significantly higher levels of COL5A2 expression in GC tissues than in normal tissues.

### Expression of COL5A2 based on scRNA-seq analysis

**Fig. 2:**
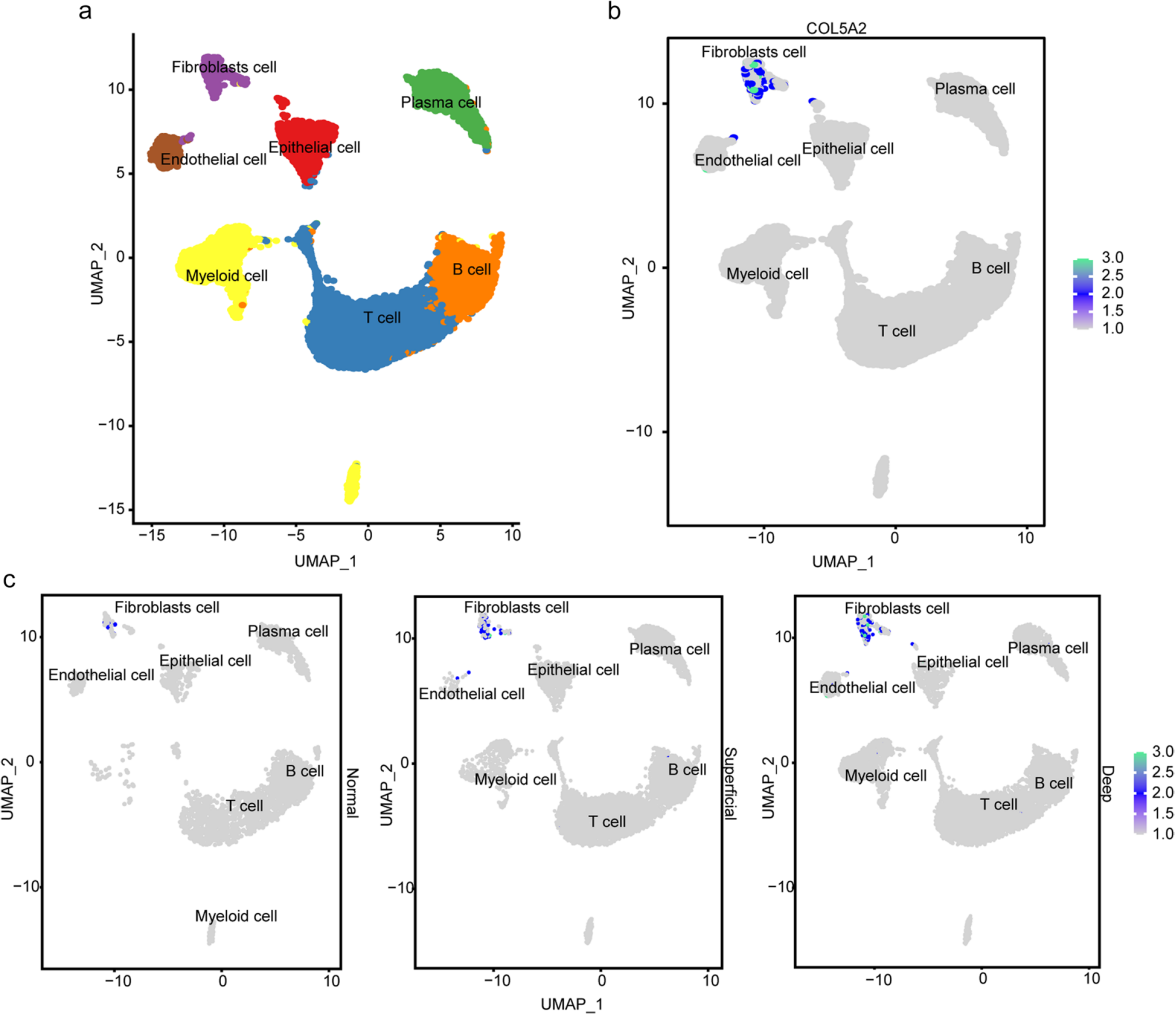
The expression of COL5A2 in cells with scRNA-seq analysis. **a** The eight major cell types in the UMAP plot. **b** COL5A2 was expressed in the fibroblasts cell type. **c** The expression of COL5A2 in normal, superficial layer and deep layer tissues

After QC, we obtained 21,909 high-quality cells, including 9854 deep layer cells, 9341 superficial layer cells and 2714 normal cells. Based on the known markers, we identified 7 kinds of cell types, including T cells (*n* = 8947), B cells (*n* = 4733), myeloid cells (*n* = 2594), plasma cells (*n* = 2591), epithelial cells (*n* = 1498), endothelial cells (*n* = 861) and fibroblasts (*n* = 685) (Fig. 2a). COL5A2 was expressed on the fibroblast cell type (Fig. 2b). There were 78 COL5A2-expressing cells in superficial layer cells, 236 COL5A2-expressing cells in deep layer cells, and only 16 COL5A2-expressing cells in normal gastric cells (Fig. 2c). It revealed that COL5A2-expressing cells were abundant in GC, and the number of cells expressing COL5A2 in the deep layer was more than that in the superficial layer (*p* < 0.05, Table 1). The results confirmed that the expression of COL5A2 was upregulated in GC and may have an association with the invasion of GC.

**Table 1:** The number of COL5A2-expressing cells in normal, superficial layer and deep layer tissue in GC with chi-square test

Comparison	p.Chisq	p.adj.Chisq
N: S	2.48e-01	2.48e-01
N: D	4.52e-09	6.78e-09*
S: D	2.70e-17	8.10e-17*

*N* Normal tissue, *S* Superficial layer tissue, *D* Deep layer tissue

^*^P < 0.05

### Relationship between COL5A2 and clinicopathological traits of GC patients

**Fig. 3:**
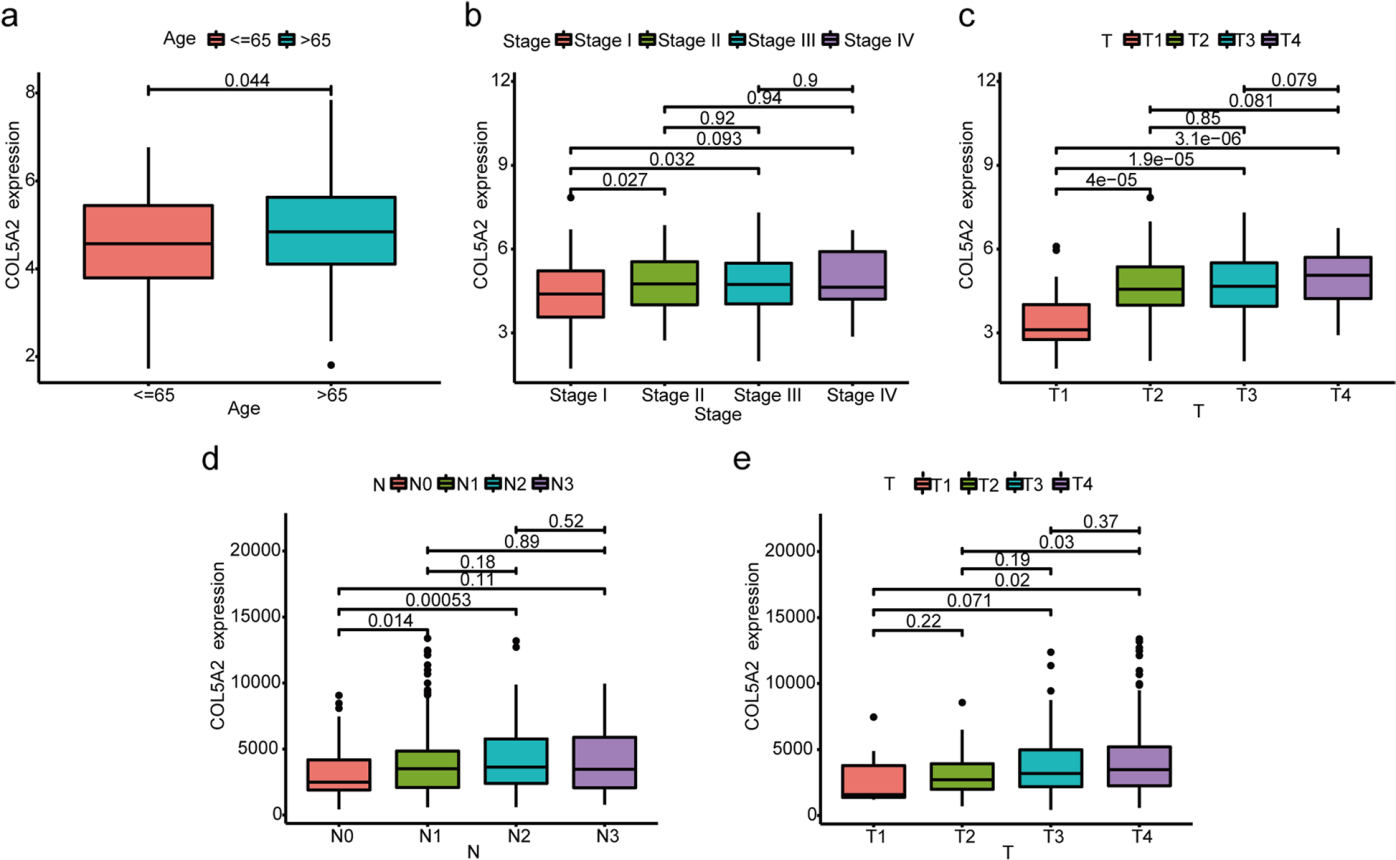
Association between COL5A2 expression and clinicopathological characteristic of gastric cancer patients. **a**-**c** The relationship between COL5A2 expression and age, stage and T classification based on TCGA data. **d**, **e** The relationship between COL5A2 expression and N and T classification based on GEO data

The association between COL5A2 expression and clinicopathological features in the TCGA clinical data was analyzed. The clinicopathological characteristics included age, gender, histologic grade, clinical stage, tumor, node and metastasis (TNM) classification, survival status and survival time (Table 2). Increased expression of COL5A2 showed significant association with age (*p* < 0.05, Fig. 3a), T classification (T1 vs. T2, T1 vs. T3, T1 vs. T4) (*P* < 0.001, Fig. 3b) and clinical stage (stageI vs. stageII, stageI vs. stageIII) (*p* < 0.05, Fig. 3c). Similarly, we verified the data in 433 additional instances in GSE84437. The upregulated COL5A2 was significantly related to T classification (T1 vs. T4, T2 vs. T4) (*P* < 0.05, Fig. 3d) and N classification (N1 vs. N0, N2 vs. N0) (N1 vs. N0 *P* < 0.05, N2 vs. N0 *P* < 0.01, Fig. 3e), according to the results. Additionally, based on IHC analysis, clinical samples were divided into low- and high-expression groups to determine the relationship between COL5A2 protein expression and clinical characteristics. As shown in Table 3, the expression of COL5A2 was significantly associated with T classification (*P* < 0.01), N classification (*P* = 0.03), and TNM stage (*P* = 0.001).

**Table 2:** Clinicopathological characteristics of gastric cancer patients from TCGA database

Clinicopathological Characteristics	Number of Gastric Cancer Patients (*N* = 443)
**Age (years)**	
≤ 65	197 (44.5%)
> 65	241 (54.4%)
Unknown	5 (1.1%)
**Gender**	
Male	285 (64.3%)
Female	158 (35.7%)
**Grade**	
G1	12 (2.7%)
G2	159 (35.9%)
G3	263 (59.4%)
Unknown	9 (2.0%)
**Stage**	
Stage I	59 (13.3%)
Stage II	130 (29.3%)
Stage III	183 (41.3%)
Stage IV	44 (9.9%)
Unknown	27 (6.1%)
**T classification**	
T1	23 (5.2%)
T2	93 (20.9%)
T3	198 (44.7%)
T4	119 (26.9%)
Unknown	10 (2.3%)
**N classification**	
N0	132 (29.8%)
N1	119 (26.9%)
N2	85 (19.2%)
N3	88 (19.9%)
Unknown	19 (4.3%)
**M classification**	
M0	391 (88.3%)
M1	30 (6.8%)
Unknown	22 (4.9%)
**Survival status**	
Death	171 (38.6%)
Survival	272 (61.4%)

**Table 3:** The expression of COL5A2 and the clinical characteristics of patients with gastric cancer

Characteristics	No. of cases (*N* = 40)	COL5A2 expression		Chi-square	*P* value
		Low	High		
**Age**				0.102	0.749
< 65	20	12	8		
≥ 65	20	11	9		
**Gender**				1.671	0.196
Male	30	15	15		
Female	10	8	2		
**Grade**				1.436	0.488
G1-G2	6	4	2		
G3	33	18	15		
Signet ring cell or mucinous	1	1	0		
**T classification**				15.920	0.000*
T1-T2	14	14	0		
T3-T4	26	9	17		
**N classification**				4.682	0.030*
N0	12	10	2		
N1-N3	28	13	15		
**M classification**				0.024	0.878
M0	39	23	16		
M1	1	0	1		
**TNM stage**				13.606	0.001*
I	8	8	0		
II	12	9	3		
III-IV	20	6	14		
**HER2 expression**				0.395	0.53
Negative	28	17	11		
Positive	12	6	6		

^*^P < 0.05 was considered significant

### COL5A2 was an independent adverse prognostic factor in GC

**Fig. 4:**
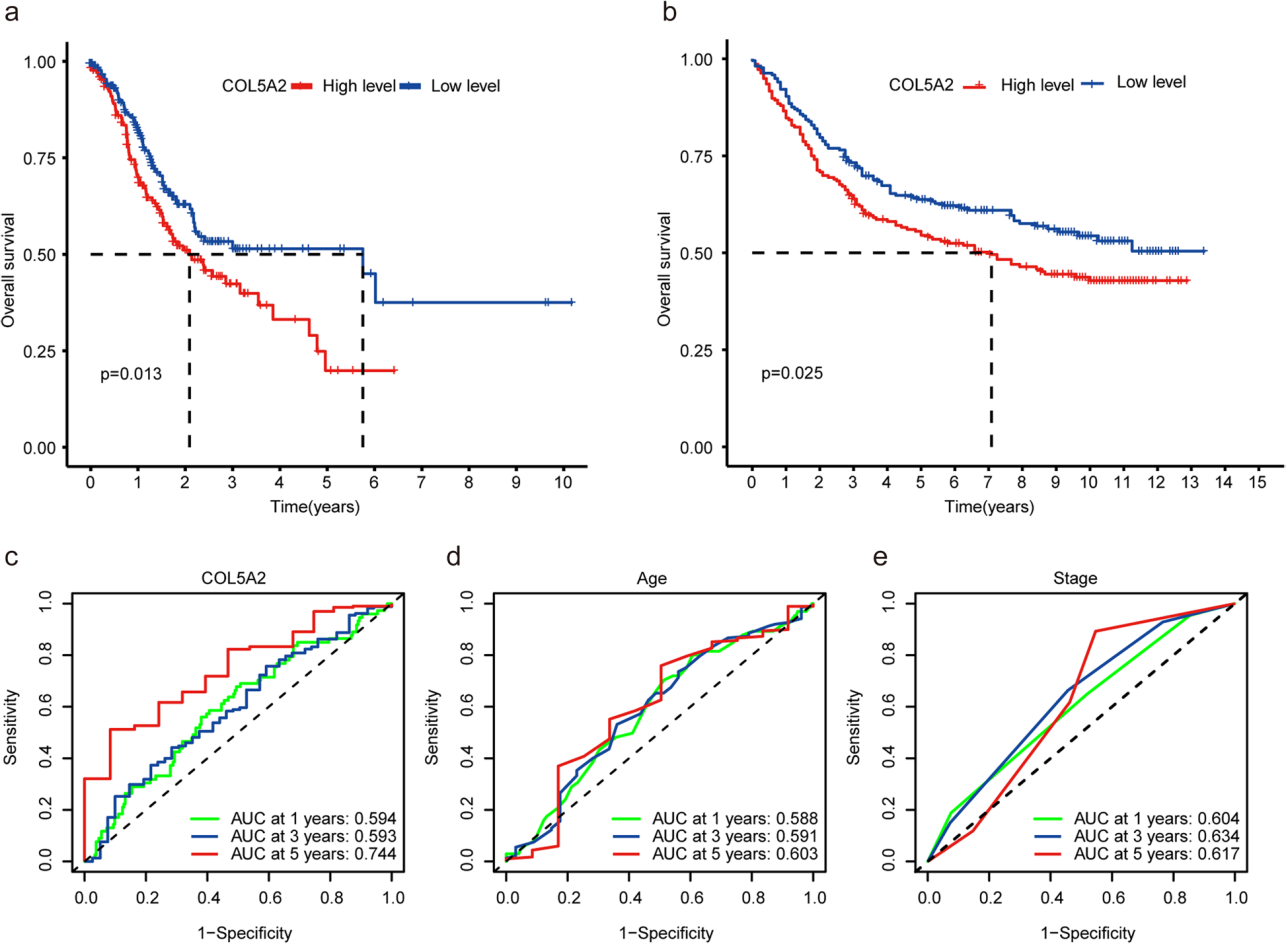
COL5A2 impact on the overall survival of patients with GC. **a** Kaplan–Meier curve was used to analyze the relationship between COL5A2 mRNA expression and prognosis of GC patients based on TCGA data (*p* = 0.013). **b** Kaplan–Meier curve was used to analyze the relationship between COL5A2 mRNA expression and prognosis of GC patients based on GEO data (*p* = 0.025). **c**-**e** Time-dependent ROC curves of patients’ OS at 1, 3, and 5 years depending on COL5A2 expression (**c**), age (**d**) and tumor stage (**e**) in the TCGA-STAD datasets

To investigate the relationship between COL5A2 expression and GC prognosis, the survival rate of high and low COL5A2 expression groups was analyzed through the Kaplan–Meier risk estimation method. Notably, survival rates were significantly lower in the group with high COL5A2 expression (*p* = 0.013, Fig. 4a). Then GSE84437 (*n* = 433) was employed to verify the result. Similarly, the high COL5A2 expression group had a worse survival rate (*p* = 0.025) (Fig. 4b). In a word, COL5A2 expression was elevated and associated with an unfavourable outcome in GC. In addition, the survival of GC was significantly associated with age, stage, T stage, N stage, and COL5A2 expression in univariate Cox regression analysis (Table 4). Age, stage, and COL5A2 expression were independent predictors of poor survival of GC in Multivariate Cox regression analysis (Table 4). Subsequently, we assessed the survival rates of GC patients on TCGA-STAD at 1, 3, and 5 years, and AUC values for time-dependent ROC curves were as follows: 0.594, 0.593, and 0.744 (Fig. 4c). The AUC value at 5 years of COL5A2 expression was higher than that of age (Fig. 4d) and tumor stage (Fig. 4e). Taken together, the above findings indicated that COL5A2 was found to be an unfavourable prognostic factor as well as an independent prognostic marker.

**Table 4:** Univariate and multivariate analysis of COL5A2 mRNA levels and clinical parameters in TCGA gastric cancer

Variables	Univariate Cox regression analysis				Multivariate Cox regression analysis			
	HR	HR.95L	HR.95H	*P*-value	HR	HR.95L	HR.95H	*P*-value
Age	1.024	1.006	1.041	0.010^**^	1.027	1.009	1.047	0.004^**^
Gender	1.403	0.959	2.054	0.081	NA	NA	NA	NA
Grade	1.330	0.941	1.883	0.107	NA	NA	NA	NA
Stage	1.549	1.245	1.929	9.13E-05^**^	1.559	1.108	2.193	0.011^*^
T	1.255	1.002	1.573	0.049^*^	0.937	0.697	1.260	0.667
M	1.806	0.971	3.358	0.062	NA	NA	NA	NA
N	1.327	1.132	1.555	4.71E-04^**^	1.116	0.907	1.374	0.301
COL5A2	1.250	1.066	1.465	5.98E-03^**^	1.211	1.026	1.430	0.024^*^

^*^P < 0.05; **P < 0.01

### COL5A2 co-expression networks in GC

**Fig. 5:**
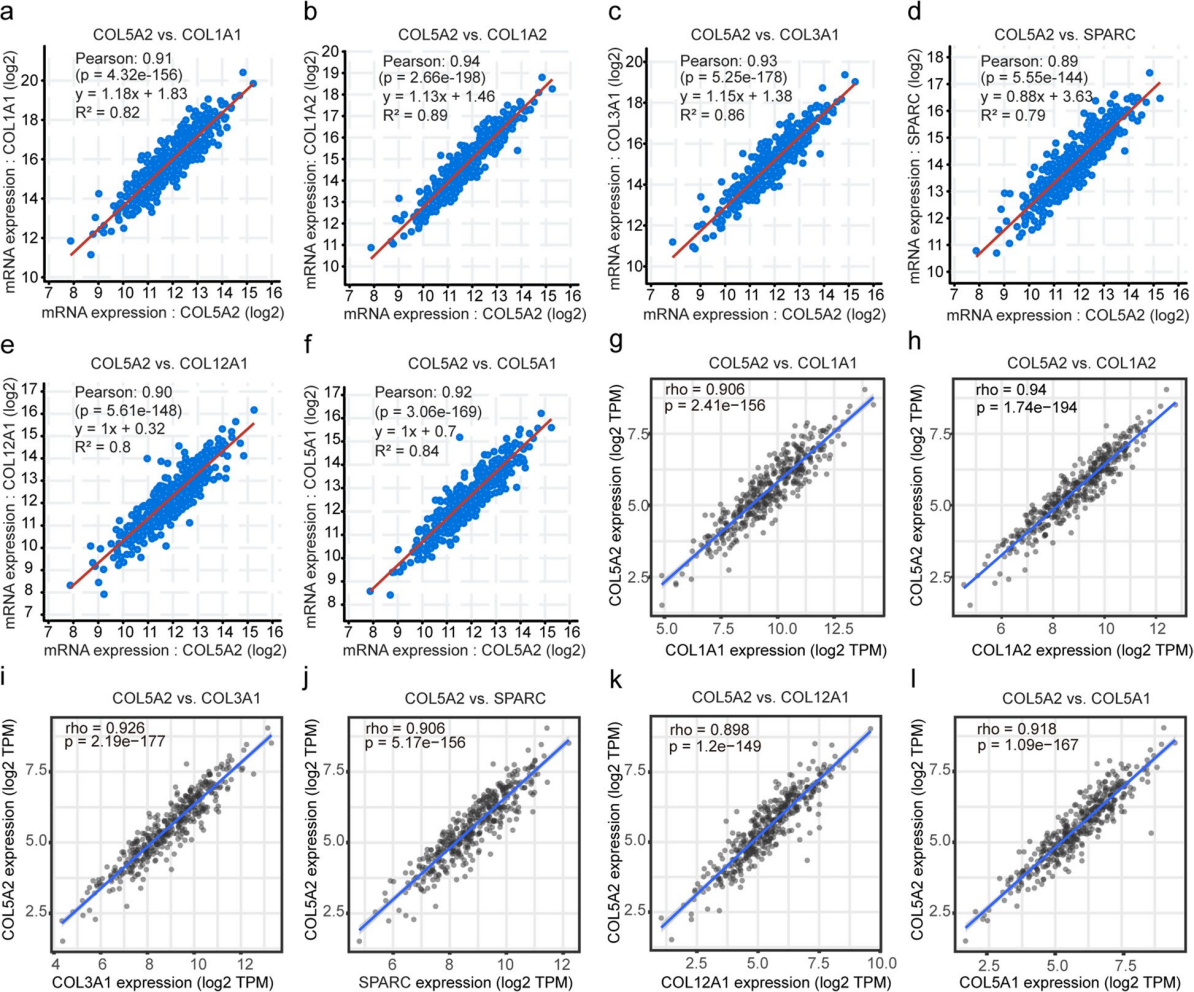
Co-expressed genes of COL5A2 in GC. **a**-**f** The genes co-expressed with COL5A2 in GC (absolute Pearson’s *r* ≥ 0.89) were assessed with the cBioPortal database. **g**-**l** COL5A2 was significantly correlated with COL1A2 (*r* = 0.94, *P* = 1.74e-194), COL3A1 (*r* = 0.926, *P* = 2.19e-177), COL5A1 (*r* = 0.918, *P* = 1.09e-167), COL1A1 (*r* = 0.906, *P* = 2.41e-156), SPARC (*r* = 0.906, *P* = 5.17e-156), COL12A1 (*r* = 0.898, *P* = 1.2e-149) in GC with the TIMER2.0 database

To gain insight into COL5A2’s biological meaning in GC, we identified genes that are positively co-expressed with COL5A2 via cBioPortal. We selected a total of six genes with high significance correlations with COL5A2 (absolute Pearson’s *r* > 0.89) for further analysis, as shown in Fig. 5a-f. Furthermore, TIMER2.0 was used to verify the correlations between COL5A2 and the six genes. According to the results, COL1A2 (*r* = 0.94, *P* = 1.74e-194), COL3A1 (*r* = 0.926, *P* = 2.19e-177), COL5A1 (*r* = 0.918, *P* = 1.09e-167), COL1A1 (*r* = 0.906, *P* = 2.41e-156), SPARC (*r* = 0.906, *P* = 5.17e-156), COL12A1 (*r* = 0.898, *P* = 1.2e-149) were strongly positively correlated with COL5A2 as shown in Fig. 5g-l. Additionally, in GC we observed a remarkable upregulation of COL5A2-related genes (Additional file 1: Figure S1). There were significant associations between these genes and GC tumor stage, except for COL12A1 (Additional file 2: Figure S2). Furthermore, COL5A2-related genes, except for COL3A1, had significant associations with poor overall survival (Additional file 3: Figure S3).

### The biological correlation of COL5A2 in GC

**Fig. 6:**
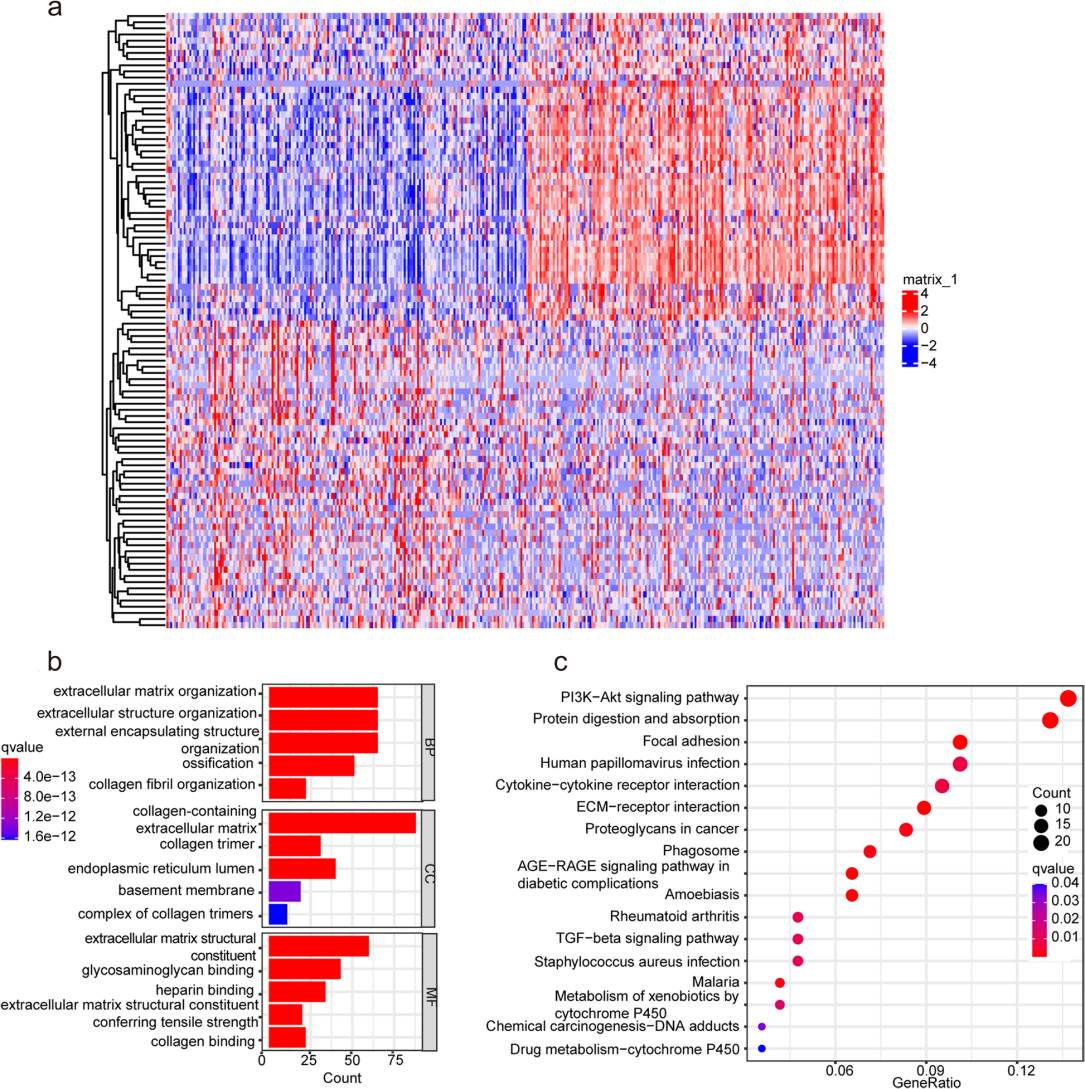
DEGs and functional analysis. **a** Heatmap of 50 DEGs. **b** GO enrichment analysis of DEGs. **c** KEGG enrichment analysis of DEGs

**Fig. 7:**
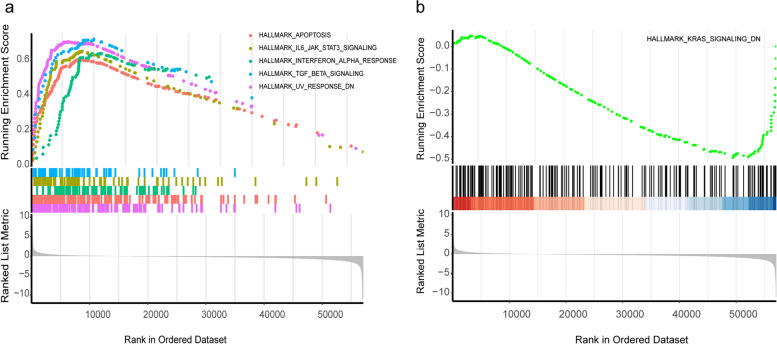
Gene set enrichment analysis of COL5A2. **a** correlation between high COL5A2 expression and hallmarks of tumor. **b** correlation between low COL5A2 expression and hallmarks of tumor

Differentially expressed genes (DEGs) analysis was employed to study COL5A2 and its potential pathogenicity in GC. We identified 521 DEGs between high and low COL5A2 expression datasets. There were 125 down-regulated genes and 396 upregulated genes among these DEGs. The 50 DEGs were shown in the heatmap (Fig. 6a). Then, the DEGs were analyzed via GO and KEGG analyses. Following were GO terms with the greatest enrichment: BP (biological process), including extracellular matrix organization, extracellular structure organization, external encapsulating structure organization, CC (cellular component) such as collagen-containing extracellular matrix, endoplasmic reticulum lumen, collagen trimer, and MF (molecular function) such as extracellular matrix structural constituent, heparin binding, glycosaminoglycan binding (Fig. 6b). Meanwhile, there were significant enrichments in the PI3K-Akt signaling pathway, focal adhesion, and protein digestion and absorption according to KEGG pathway analysis (Fig. 6c). Since COL5A2 expression was related to tumor stage as well as the prognosis of GC, we hypothesised that upregulated COL5A2 could promote tumor progression. Moreover, GSEA was conducted and found that there was a dynamic correlation between high COL5A2 expression and hallmarks of tumor such as “angiogenesis”, “IL6-JAK-STAT3 signaling” and “NOTCH signaling”. As opposed to “KRAS signaling Dn”, there was a significant enrichment in the low COL5A2 expression group (Fig. 7a, b). The results may provide a potential mechanism of tumor progression.

### COL5A2 affects infiltrating immune cells in GC

**Fig. 8:**
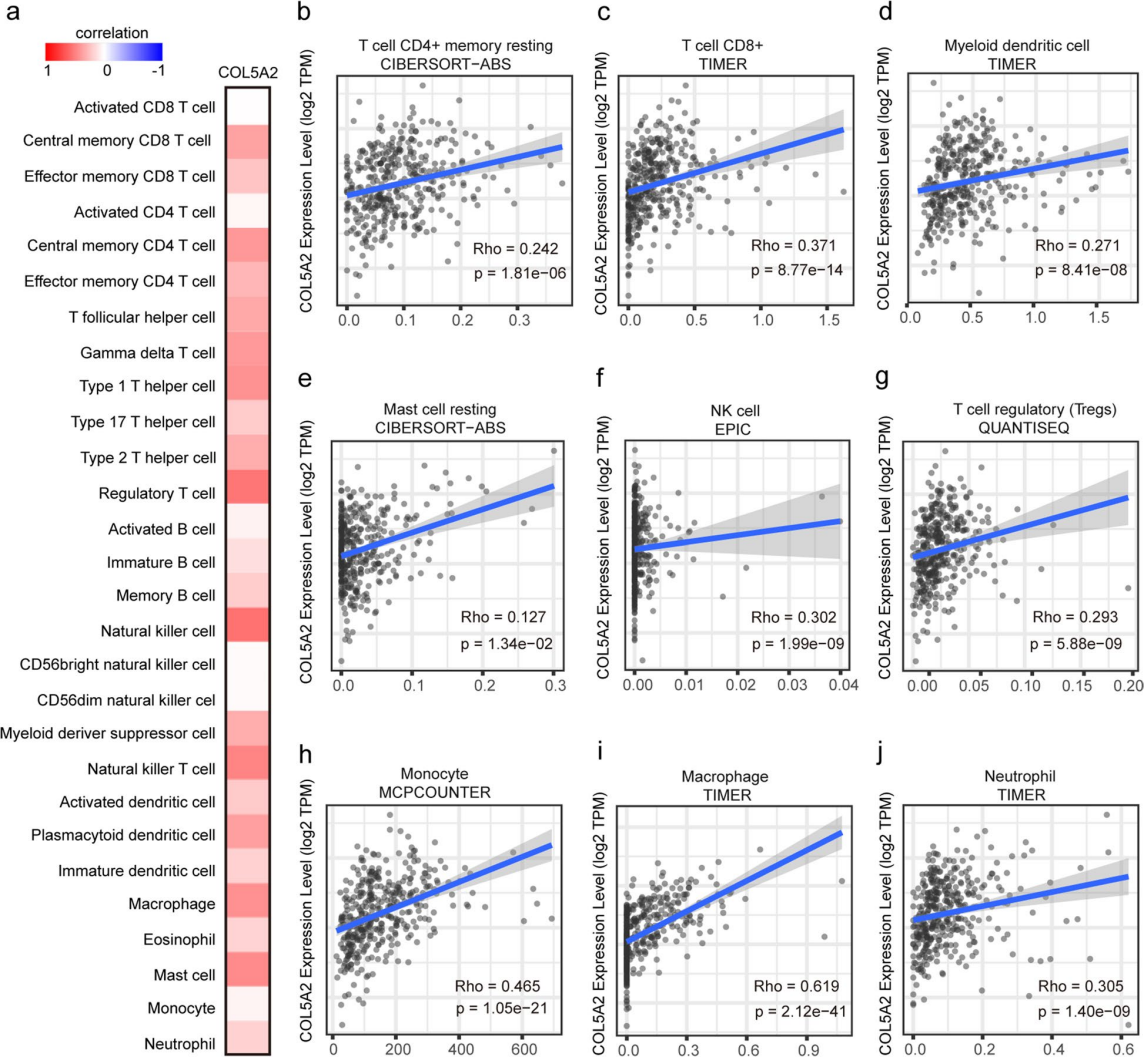
Correlation between COL5A2 expression and immune infiltration in GC. **a** Correlation between the expression of COL5A2 and the abundance of TILs in GC available at TISIDB database. **b**-**j** Correlation of COL5A2 expression with infiltration levels of CD8 + T cell, CD4 + T cell, Treg cell, mast cell resting, neutrophil, macrophage, dendritic cell, natural killer cell, and monocyte in gastric cancer available at TIMER2.0 database

Tumor progression, treatment responses, and prognosis are affected by immune cell infiltration. Hence, an analysis of the relationship between immune cell infiltration and COL5A2 expression in GC was conducted via TISIDB as well as TIMER2.0. There was a significant association between COL5A2 expression and the abundance of TILs, as revealed by the results (Fig. 8a). For example, COL5A2 expression was positively correlated with the infiltrating levels of central memory CD8 T cell (rho = 0.359, *p* = 6.03e-14), central memory CD4 T cell (rho = 0.403, *p* < 2.2e-16), Th1 (rho = 0.428, *p* < 2.2e-16), Treg (rho = 0.538, *p* < 2.2e-16), NK (rho = 0.555, *p* < 2.2e-16), plasmacytoid dendritic cell (pDC) (rho = 0.377, *p* = 1.59e-15), macrophage (rho = 0.437, *p* < 2.2e-16), mast cell (rho = 0.454, *p* < 2.2e-16). In addition, we employed EPIC, QUANTISEQ, COUNTER, CIBERSORT-ABS, TIMER and XCELL tools from TIMER2.0 to confirm the relationship between immune cell infiltration and COL5A2 expression (Fig. 8b-j). As a result, COL5A2 expression affected the levels of immune infiltration.

### COL5A2 expression was associated with immune marker sets

To investigate the correlation between COL5A2 expression and multifarious immune infiltrating cells, the relationship between COL5A2 expression and immune marker sets of diverse immune cells of STAD was analyzed in the TIMER2.0 and GEPIA databases. The immune cells included T cells (general), CD8 + T cells, monocytes, M1 and M2 macrophages, B cells, TAMs, NK cells, DCs and neutrophils. Moreover, we examined functional T cells, such as Th1, Th2, Treg, Th17, Tfh and exhausted T cells. Using purity-adjusted correlations, a link between COL5A2 expression and immune cells can only be established when COL5A2 was associated with all immune cells’ markers. we found that COL5A2 expression was significantly related to M1 and M2 macrophages, TAMs, monocytes, Th1, Th2, Treg, and T cell exhaustion (Table 5). Furthermore, correlations between COL5A2 expression and immune markers of monocytes, M1 and M2 macrophages, and TAMs were examined via GEPIA. As with TIMER2.0, the results were similar (Additional file 4: Table S1). All the above findings indicated that COL5A2 expression affected immune infiltration levels in GC.

**Table 5:** Correlation analysis between COL5A2 and immune marker sets of immune cells in TIMER2.0

Description	Gene markers	STAD			
		None		Purity	
		Cor	*P*	Cor	*P*
CD8 + T cell	CD8A	0.132	*	0.106	0.0392
	CD8B	0.001	0.977	-0.014	0.78
T cell (general)	CD3D	0.098	0.0454	0.066	0.202
	CD3E	0.013	0.0359	0.067	0.195
	CD2	0.179	**	0.156	*
B cell	CD19	0.023	0.634	0.013	0.805
	CA79A	0.039	0.432	0.002	0.969
Monocyte	CD86	0.45	***	0.436	***
	CA115(CSF1R)	0.451	***	0.438	***
TAM	CCL2	0.394	***	0.368	***
	CD68	0.31	***	0.285	***
	IL10	0.433	***	0.423	***
M1 Macrophage	INOS (NOS2)	0.128	*	0.138	*
	IRF5	0.216	***	0.214	***
	COX2(PTGS2)	0.385	***	0.39	***
M2 Macrophage	CD163	0.513	***	0.489	***
	VSIG4	0.497	***	0.484	***
	MS4A4A	0.451	***	0.441	***
Neutrophils	CD66b (CEACAM8)	0.012	0.811	0.034	0.507
	CD11b (ITGAM)	0.447	***	0.435	***
	CCR7	0.107	0.0295	0.079	0.127
Natural killer cell	KIR2DL1	0.133	*	0.138	*
	KIR2DL3	0.121	0.0136	0.124	0.0159
	KIR2DL4	0.086	0.0813	0.053	0.305
	KIR3DL1	0.096	0.0508	0.076	0.137
	KIR3DL2	0.126	*	0.12	0.0191
	KIR3DL3	-0.038	0.437	-0.023	0.655
	KIR2DS4	0.106	0.031	0.092	0.0726
Dendritic cell	HLA-DPB1	0.16	*	0.13	0.0114
	HLA-DQB1	0.107	0.0299	0.078	0.13
	HLA-DRA	0.167	**	0.146	*
	HLA-DPA1	0.148	*	0.121	0.0186
	BDCA-1(CD1C)	0.092	0.0609	0.07	0.175
	BDCA-4(NRP1)	0.606	***	0.592	***
	CD11c (ITGAX)	0.492	***	0.497	***
Th1	T-bet (TBX21)	0.15	*	0.136	*
	STAT4	0.234	***	0.223	***
	STAT1	0.218	***	0.211	***
	IFN-γ (IFNG)	0.119	0.0151	0.11	0.0316
	TNF-α (TNF)	0.214	***	0.194	**
Th2	GATA3	0.172	**	0.157	*
	STAT6	0.126	*	0.127	0.0131
	STAT5A	0.351	***	0.354	***
	IL13	0.138	*	0.179	**
Tfh	BCL6	0.315	***	0.279	***
	IL21	0.088	0.0744	0.074	0.152
Th17	STAT3	0.4	***	0.389	***
	IL17A	-0.04	0.422	-0.051	0.319
Treg	FOXP3	0.216	***	0.248	***
	CCR8	0.361	***	0.362	***
	STAT5B	0.361	***	0.367	***
	TGFβ (TGFB1)	0.508	***	0.498	***
T cell exhaustion	PD-1 (PDCD1)	0.131	*	0.123	0.0163
	CTLA4	0.2	***	0.19	**
	LAG3	0.15	*	0.121	0.0189
	TIM-3 (HAVCR2)	0.482	***	0.471	***
	GZMB	0.199	***	0.166	*

*STAD* Stomach adenocarcinoma, *TAM* Tumor-associated macrophage

^*^P < 0.01; **P < 0.001; ***P < 0.0001

### Expression of COL5A2 was associated with immune checkpoint genes

**Fig. 9:**
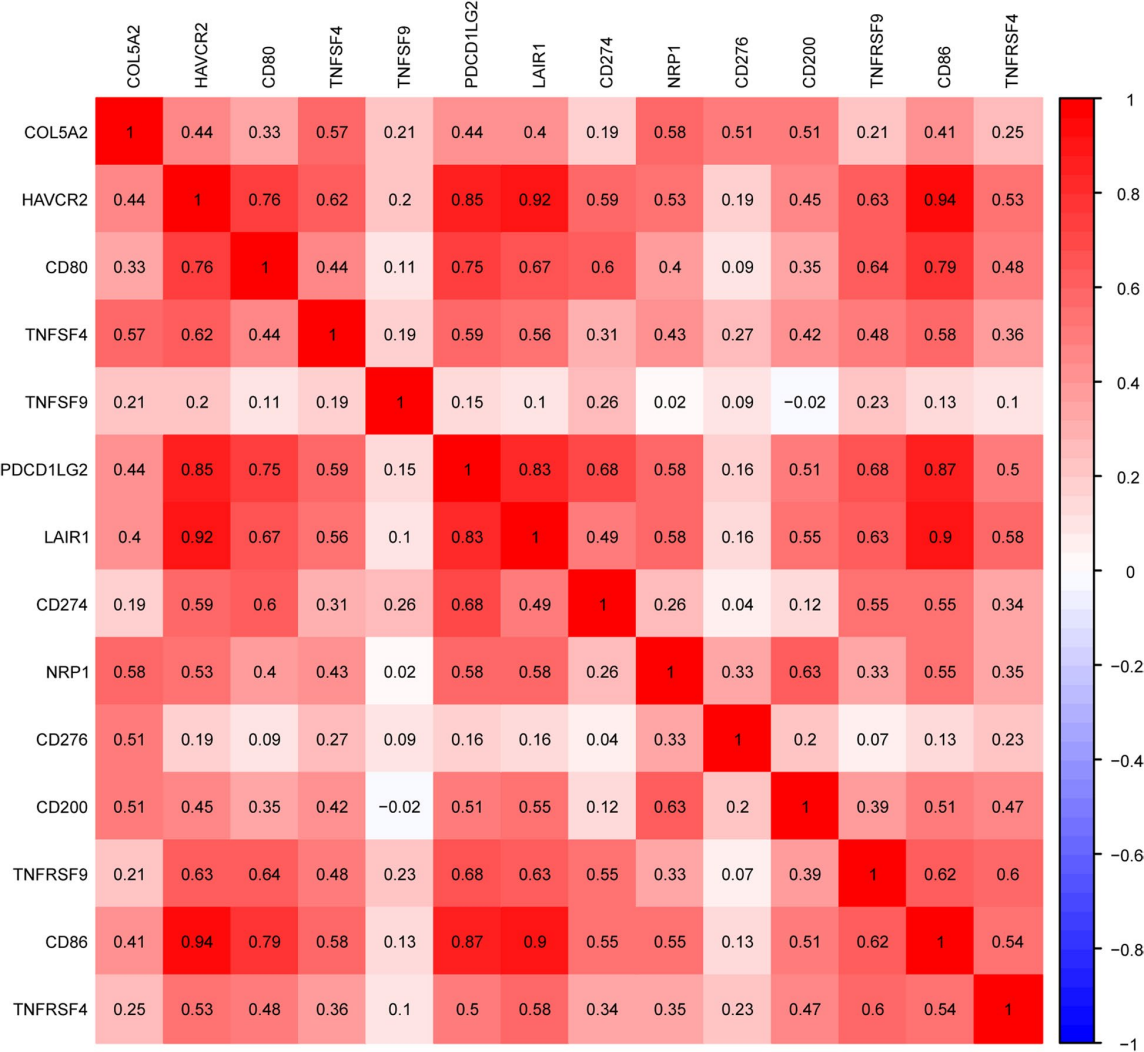
Correlation analysis of COL5A2 with those of 22 common immune checkpoint genes in gastric cancer

It is possible to impair the antitumor immune response by overexpressing immune checkpoint molecules. This can cause tumor immune escape and lead to tumor progress [27, 28]. Hence, immune checkpoint molecules were analyzed for their association with COL5A2 expression. According to the results, in GC, many immune checkpoint molecules, such as CD276, PDCD1LG2, HAVCR2, CD200, and CD274 were positively correlated with COL5A2 (Fig. 9). These findings suggested that high COL5A2 expression may cause immune tolerance and escape through multiple immune checkpoint pathways, and lead to worse outcomes for patients with GC. However, the discoveries deserve further clinical verification.

### The ability of COL5A2 expression to predict immunotherapy sensitivity

**Fig. 10:**
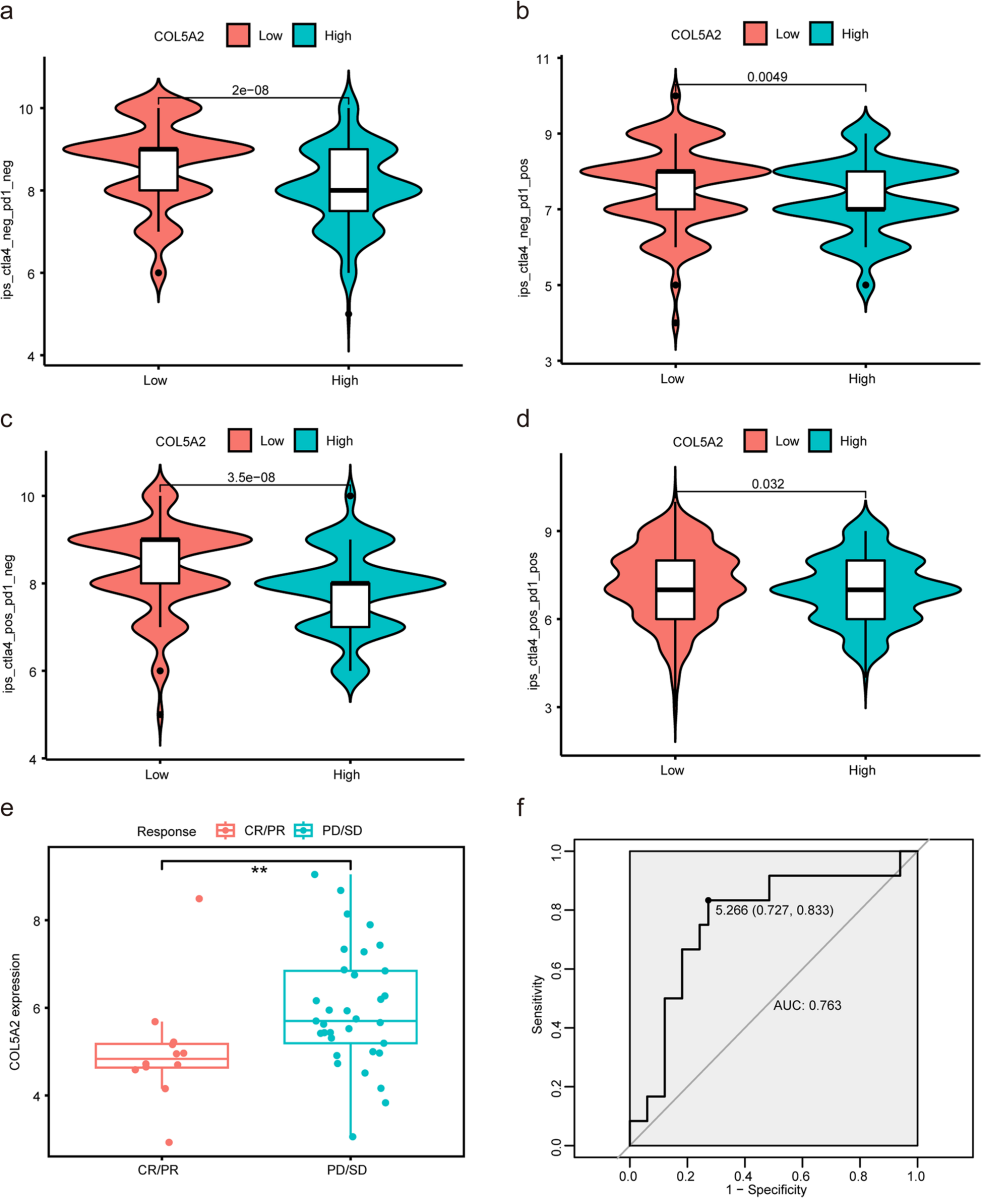
Correlation between COL5A2 expression and sensitivity to immunotherapy. **a**-**d** The IPS in high and low expression groups of COL5A2. **e** Comparison of COL5A2 expression between responders and non-responders to immunotherapy in PRJEB25780 cohort. **f** ROC curve for COL5A2 expression in predicting response to immunotherapy. **p < 0.01; CR, complete response; PR, partial response; SD, stable disease; PD, progressive disease

Nowadays, immunotherapy is a main option for cancer therapy [29, 30], particularly immune checkpoint blockade (ICB) targeting cytotoxic T-lymphocyte-associated antigen 4 (CTLA-4) and PD-1 [31]. Based on COL5A2 expression, we assessed the immunogenicity of each group via IPS analysis. As a result, low COL5A2 expression was linked to higher scores for ips_ctla4_pos_pd1_pos (CTLA-4 positive response and PD-1 positive response), ips_ctla4_neg_pd1_neg (CTLA-4 negative response and PD-1 negative response), ips_ctla4_pos_pd1_neg, as well as ips_ctla4_neg_pd1_pos (Fig. 10a-d). Furthermore, we validated the ability of COL5A2 expression to predict the immunotherapy response with the datasets, which included 45 advanced GC patients treated with pembrolizumab monotherapy. Results showed that patients responding to pembrolizumab expressed significantly lower levels of COL5A2 than those who did not (*p* < 0.01, Fig. 10e). AUC of 0.763 was obtained for COL5A2 expression level predicting the response of gastric cancer patients to pembrolizumab (Fig. 10f). Hence, the patients with low COL5A2 expression may benefit more from CTLA-4 or anti-PD-1 immunotherapy, and COL5A2 expression may be used to predict the immunotherapy response.

## Discussion

Collagens, the main protein in the ECM, have been widely reported in diverse cancers [32]. The migration of GC cells was promoted when COL5A2 was overexpressed and inhibited when COL5A2 was silenced in vitro and in vivo [33]. According to previous researchers, COL5A2 was overexpressed in GC via RT-qPCR and immunohistochemistry methods and was related to GC prognosis and renal metastasis [34]. A COL3A1/FBN1/COL5A2/SPARC-mir-29a-3p-H19 ceRNA network in GC was revealed by Shen et al. as a prognostic indicator [35]. Mao et al. according to the expression of M2 macrophages, COL5A2, EGF and FLT1 together with clinicopathological characteristics established a nomogram which was an important factor in determining prognosis [36]. Nevertheless, Cao et al. [37] showed that COL5A2 was not related to lower overall survival in GEO datasets. Therefore, the effect of COL5A2 expression on GC is worth to be further explored. In this study, we found that COL5A2 was overexpressed in GC. Based on the single-cell sequencing analysis, a spatial gradient of COL5A2 expression was observed in diffuse-type gastric cancer. Additionally, the high expression of COL5A2 was associated with advanced TNM stage, patients’ prognosis and immune infiltration, and may be used as a predictor of the effect of immunotherapy. Therefore, COL5A2 may promote the invasiveness of GC and may serve as a biomarker for GC prognosis and treatment.

The functions of co-expressed genes are generally similar. The co-expressed analysis revealed that the top six co-expressed genes, including COL1A2, COL3A1, COL5A1, COL1A1, SPARC and COL12A1, had a strong positive correlation with COL5A2. Most of them were ECM molecules. They were all upregulated in GC, and the majority of them served as risk factors for advanced stage and poor survival of GC. Co-expressed genes have been shown to promote tumor progression or worse survival in cancers [38–43]. Taken together, we speculated that COL5A2 and its co-expressed genes might be good prognosis markers of GC.

Functional enrichment analyses including GO and KEGG enrichment analyses showed that COL5A2 primarily regulated ECM- related processes, PI3K-AKT signaling pathway and focal adhesion. Normal tissue phenotype and homeostasis are regulated by the ECM, and dysfunction of ECM promotes the development of the tumor [44]. Focal adhesions, connect points between cells and the extracellular matrix [45] and provide a necessary site for adhesion to the matrix during cancer cell migration [46]. Besides, PI3K/AKT signaling pathway is an important factor contributing to tumor progression and poor survival of GC [47, 48]. In addition, GSEA analyses revealed that in the high-COL5A2 expression group, tumor progression-related pathways, including angiogenesis, IL6-JAK-STAT3 signaling as well as Notch signaling were enriched. The occurrence, development, and tumor characteristics of GC are influenced by these signaling pathways. The IL6-JAK-STAT3 pathway, for instance, plays an important role in the pathogenesis of various human malignancies [49]. There is an association between STAT3 and the advanced TNM stage as well as an unfortunate outcome of GC [50]. The Notch signaling pathway also takes participate in regulating many aspects of cancer biology and regulates the crosstalk between the different compartments of the tumor microenvironment (TME) [51]. In GC, NOTCH3 contributes to immune tolerance and promotes tumor development [52]. These results revealed that COL5A2 acted as a regulator of key functions and signaling pathways in GC, leading to an unfortunate prognosis.

Fibroblasts, endothelial cells, immune cells, and their secreted ECM form TME [53]. TME contributes to the progression of tumors and can reduce the resistance of tumor cells to chemotherapy and immunotherapy [54]. The TME and tumor cells may interact through epithelial-mesenchymal transition (EMT), which is an important regular of tumor metastasis [55]. It reported that EMT processes and the immunosuppressive microenvironment in gliomas are regulated by COL5A2 [56]. In addition, the tumor-associated ECM can regulate immune effects as well as the migration and localization of immune cells [57, 58]. COL5A2 encodes extracellular proteoglycans, glycoproteins, and other elements of ECM, contributing to determining ECM composition [59]. Therefore, for GC immunotherapy, COL5A2 could be a possible target.

Immune cells infiltrating tumor tissues not only disrupt cytokine signals but also play an important role in cancer biology [60]. In this study, the association between COL5A2 expression with immune infiltration levels in GC was analyzed. It revealed that COL5A2 expression was positively related to immune infiltration levels, such as central memory CD8 and CD4 T cells, Th1, Treg, NK, pDC, macrophages, and mast cells. In addition, we found that COL5A2 expression correlated with immune cell marker genes, which indicated that COL5A2 was involved in regulating tumor immunology. Firstly, COL5A2 expression was weakly correlated with macrophage M1 markers like inducible nitric oxide synthase (iNOS) (NOS2) and interferon regulatory factor 5 (IRF5), while strongly correlated with macrophage M2 gene markers. The findings indicated that COL5A2 may be responsible for polarizing TAMs. The majority of TAMs exhibit the M2 phenotype, which indicates poor outcomes in solid tumors in TME [61]. It has been shown that M2 macrophages can contribute to GC progression and metastasis [62]. Therefore, COL5A2 expression may be related to immunosuppressive activity in GC. Secondly, COL5A2 expression was positively correlated with Treg and T cell exhaustion gene markers. One of the reasons for immune escape is the increase in Tregs in the TME [63]. FOXP3 + Tregs suppress abnormal immune responses against self-antigens and maintain immune homeostasis [64]. Tim3 and PD-1 control T cell responses and are closely linked to T cell exhaustion [65, 66], which is characterized by poor proliferation, reduced cytotoxicity, and loss of effector function [67]. Accordingly, COL5A2 may promote immunosuppression by enhancing Treg differentiation and T cell exhaustion. Thirdly, COL5A2 expression showed significant correlations with the cell markers of T helper cells (Th1, Th2). It may be the possible mechanism that COL5A2 regulates the function of T cells. These results all together revealed that COL5A2 expression may impact immune responses by influencing immunocytes infiltration in GC immune microenvironment, which contributes to a worse prognosis.

Immune checkpoints act on stimulatory and inhibitory pathways and are essential to maintain self-tolerance and regulate the type, magnitude, and duration of immune responses [68]. As a result of activating immune checkpoint pathways, tumors prevent the immune system from recognizing them, thereby inhibiting the immune response [69]. We found a positive correlation between COL5A2 expression and immune checkpoint inhibitors. This indicated that high COL5A2 expression may promote the immune escape of tumor cells in GC. Nevertheless, research is needed to understand the mechanism by which COL5A2 interacts with immune checkpoint molecules.

By blocking immune-regulatory receptors like PD-1 and CTLA-4, T cells are restored to function and proliferation, thereby reinvigorating the antitumor immune response [68]. Even though cancer immunotherapy has advanced considerably, most patients do not respond to or benefit from ICB treatment. Immune biomarkers are also important factors that influence the immunotherapy response [70]. Hence, a well-effect of clinically personalized immunotherapy needs comprehensive and precision pre-biomarkers. Considering that COL5A2 expression affects TME immune cell infiltration and may promote tumor cell immune escape, we further analyzed whether COL5A2 expression could assess the response to immunotherapy. Our study demonstrated that COL5A2 expression level may be used to identify GC patients’ sensitivity to PD-1 or CTLA-4. Given the associations between COL5A2 expression and the immune landscape, a greater understanding of the mechanisms of COL5A2 for the development of effective treatment strategies is crucial.

Nevertheless, limitations remain in our study. The analysis bias caused by the present retrospective study is inevitable. We will be consistent to perform forward-looking studies further to avoid bias. In addition, to investigate and certify the character and regulatory mechanisms of COL5A2, further experimental studies in vivo and in vitro are necessary.

## Conclusions

COL5A2 was upregulated in GC, and the expression had a spatial gradient. COL5A2 had an association with clinicopathological characteristics and terrible outcomes in GC. Additionally, in GC patients, COL5A2 was an independent prognostic factor. Moreover, we also found that COL5A2 expression may be related to immune mechanisms and may be used to predict the clinical immunotherapy response. Accordingly, the results suggested that COL5A2 acted as an important role in GC and might be able to contribute to GC immunotherapy.

### Supplementary Information

**Additional file 1: Figure S1.** Analysis of the expression of COL5A2 co-expressed genes in gastric cancer using GEPIA. (a-f) the expression of COL1A2, COL3A1, COL5A1, COL1A1, SPARC and COL12A1 mRNA levels in GC tissues and normal gastric tissues. *, *P*<0.05.

**Additional file 2: Figure S2.** Analysis of the correlations between COL5A2 co-expressed genes and gastric cancer stage using GEPIA. (a-f) The association between COL1A2, COL3A1, COL5A1, COL1A1, SPARC and COL12A1 expression and gastric cancer stage.

**Additional file 3: Figure S3.** Analysis of the correlations between COL5A2 co-expressed genes and the survival of gastric cancer patients using Kaplan-Meier plotter online database. (a-f) The association between COL1A2, COL3A1, COL5A1, COL1A1, SPARC and COL12A1 expression and gastric cancer survival.

**Additional file 4: Table S1.** Analysis of the correlations between COL5A2 expression and immune marker sets through GEPIA.

## Data Availability

The data generated and analyzed during this study are described in the following data record: 10.6084/m9.figshare.23647089 [72]. The datasets used and analyzed during the current study are available from the cancer genome database (TCGA-STAD) https://portal.gdc.cancer.gov and NCBI Gene Expression Omnibus (GEO: GSE84437) https://www.ncbi.nlm.nih.gov/geo/. Single-cell RNA sequencing (scRNA-seq) data GSE167297 was downloaded from GEO https://www.ncbi.nlm.nih.gov/geo/query/acc.cgi?acc=GSE167297. RNA-Seq data PRJEB25780 were downloaded from ENA (https://www.ebi.ac.uk/ena/data/view/PRJEB25780). These data are free and publicly available.

## Abbreviations

GC: Gastric cancer
COL5A2: Collagen type V alpha 2
TCGA: The cancer genome database
STAD: Stomach adenocarcinoma
GEO: Gene Expression Omnibus
ENA: European Nucleotide Archive
IPS: Immunophenoscore
scRNA-seq: Single-cell RNA sequencing
QC: Quality control
PCA: Principal components
UMAP: Uniform Manifold Approximation and Projection
DEGs: Differential expression genes
AUC: Area under the receiver operating characteristic curve
ROC: Receiver operating characteristic
BP: Biological process(s)
GO: Gene Ontology
CC: Cellular component
MF: Molecular function
KEGG: Kyoto Encyclopedia of Genes and Genomes
GSEA: Gene Set Enrichment Analysis
OS: Overall survival
ECM: Extracellular matrix
IPS: Immunophenoscore
TIMER2.0: Tumor Immune Estimation Resource 2.0
FFPE: Formalin-fixed paraffin-embedded
TILs: Tumor-infiltrating lymphocytes
TAMs: Tumor-associated macrophages
DCs: Dendritic cells
pDC: Plasmacytoid dendritic cell
NK: Natural killer
Th: T helper cell
Tfh: Follicular helper T cell
Treg: Regulatory T cell
GEPIA: The Gene Expression Profiling Interactive Analysis
TCIA: The Cancer Immunome Atlas
ICB: Immune checkpoint blockade
CTLA-4: Cytotoxic T-lymphocyte-associated antigen 4
PD-1: Programmed cell death protein 1
CR: Complete response
PR: Partial response
SD: Stable disease
PD: Progressive disease
EMT: Epithelial-mesenchymal transition
iNOS: Inducible nitric oxide synthase
IRF5: Interferon regulatory factor 5
