# Supplementary figures and images for: COL5A2 is a prognostic-related biomarker and correlated with immune infiltrates in gastric cancer based on transcriptomics and single-cell RNA sequencing
Source: BMC Med Genomics. 2023 Sep 18;16:220. doi: 10.1186/s12920-023-01659-9 (PMC10506210; doi:10.1186/s12920-023-01659-9)

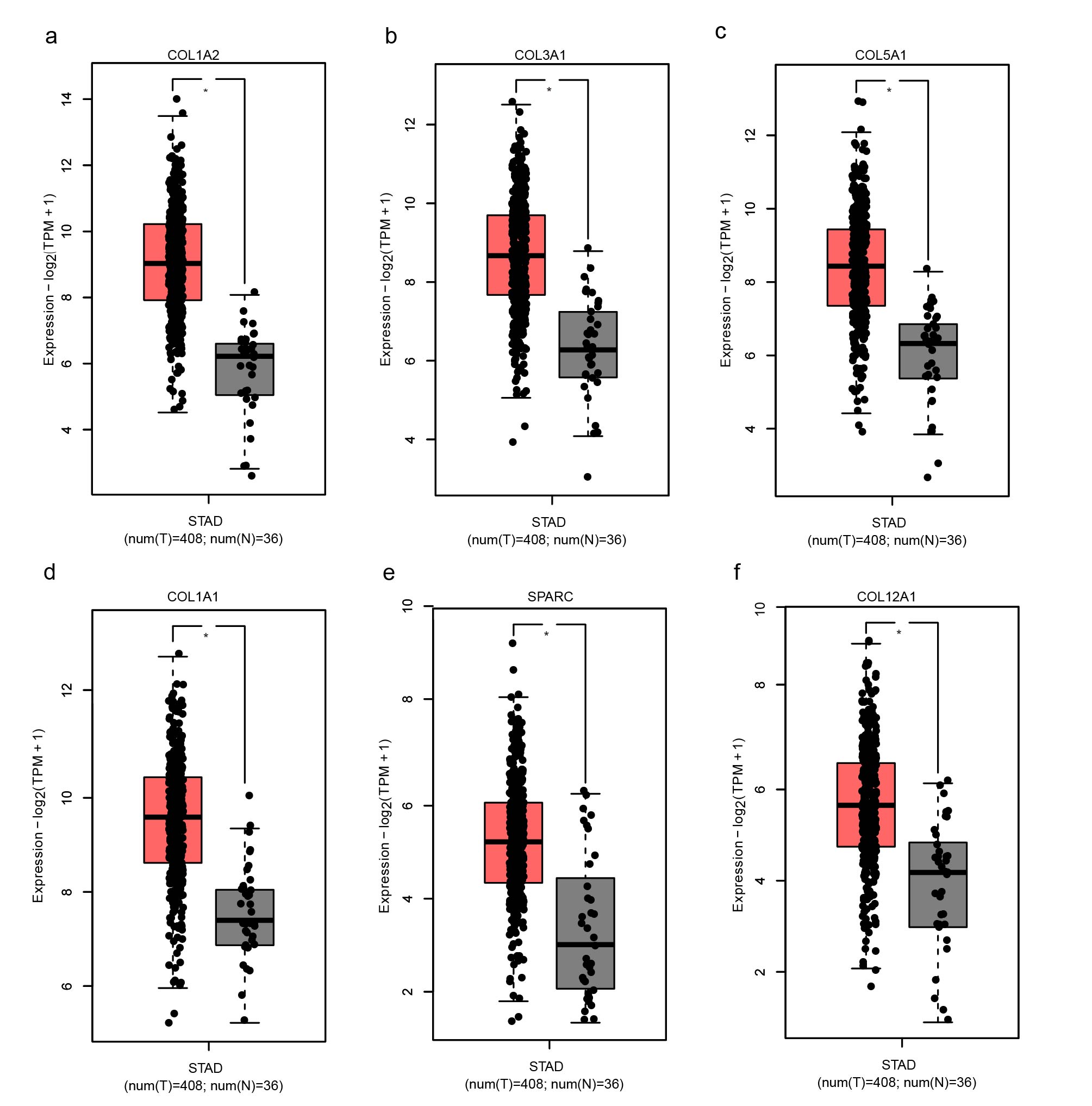

Supplement: Supplementary file 1 — Additional file 1: Figure S1. Analysis of the expression of COL5A2 co-expressed genes in gastric cancer using GEPIA. (a-f) the expression of COL1A2, COL3A1, COL5A1, COL1A1, SPARC and COL12A1 mRNA levels in GC tissues and normal gastric tissues. *, P<0.05. [file 12920_2023_1659_MOESM1_ESM.jpg]

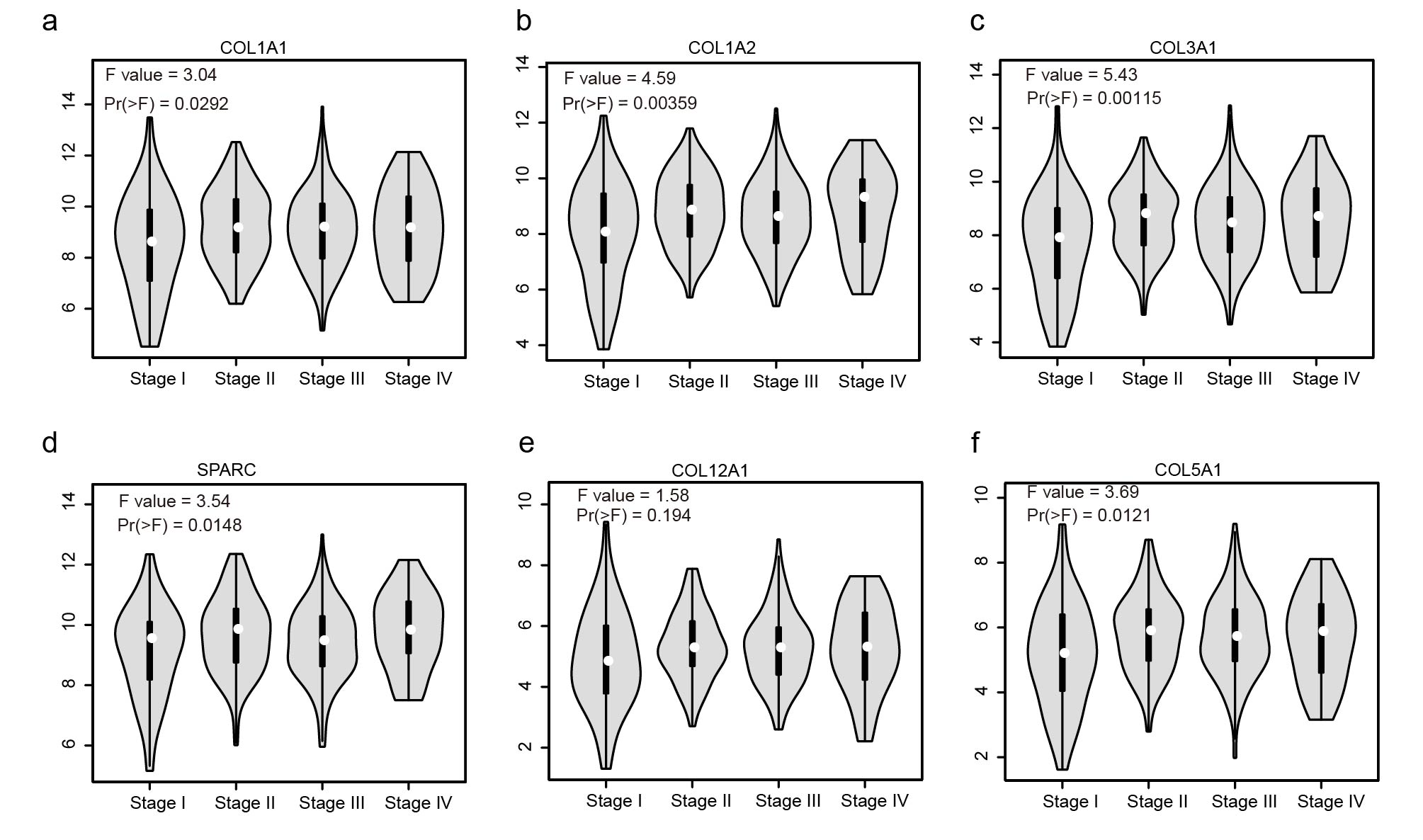

Supplement: Supplementary file 2 — Additional file 2: Figure S2. Analysis of the correlations between COL5A2 co-expressed genes and gastric cancer stage using GEPIA. (a-f) The association between COL1A2, COL3A1, COL5A1, COL1A1, SPARC and COL12A1 expression and gastric cancer stage. [file 12920_2023_1659_MOESM2_ESM.jpg]

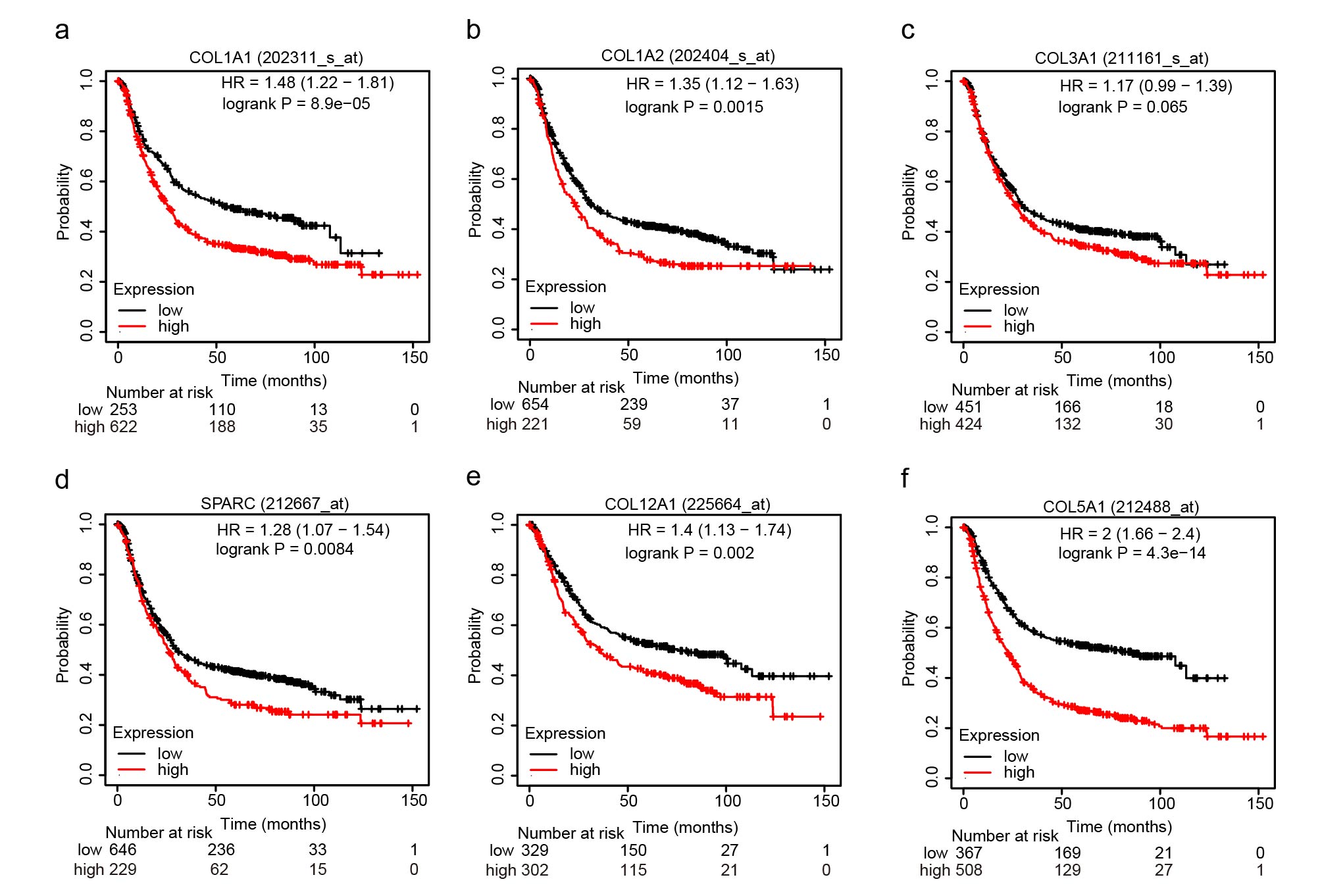

Supplement: Supplementary file 3 — Additional file 3: Figure S3. Analysis of the correlations between COL5A2 co-expressed genes and the survival of gastric cancer patients using Kaplan-Meier plotter online database. (a-f) The association between COL1A2, COL3A1, COL5A1, COL1A1, SPARC and COL12A1 expression and gastric cancer survival. [file 12920_2023_1659_MOESM3_ESM.jpg]
