# Supplementary material for: COL5A2 is a prognostic-related biomarker and correlated with immune infiltrates in gastric cancer based on transcriptomics and single-cell RNA sequencing
Source: BMC Med Genomics. 2023 Sep 18;16:220. doi: 10.1186/s12920-023-01659-9 (PMC10506210; doi:10.1186/s12920-023-01659-9)
Supplement: Supplementary file 4 — Additional file 4: Table S1. Analysis of the correlations between COL5A2 expression and immune marker sets through GEPIA. [file 12920_2023_1659_MOESM4_ESM.docx]

Table S1 Correlation analysis between COL5A2 expression and immune markers of monocyte, macrophages and TAM in GEPIA

| Description | Gene markers | STAD | | | |
| --- | --- | --- | --- | --- | --- |
|  |  | Tumor | | Normal | |
|  |  | R | P | R | P |
| Monocyte | CD86 | 0.45 | *** | -0.053 | 0.76 |
|  | CA115(CSF1R) | 0.48 | *** | 0.34 | 0.044 |
| TAM | CCL2 | 0.41 | *** | 0.34 | 0.044 |
|  | CD68 | 0.34 | *** | -0.4 | 0.016 |
|  | IL10 | 0.46 | *** | 0.12 | 0.5 |
| M1 Macrophage | INOS (NOS2) | 0.13 | * | 0.21 | 0.23 |
|  | IRF5 | 0.27 | *** | -0.42 | 0.01 |
|  | COX2(PTGS2) | 0.4 | *** | 0.66 | *** |
| M2 Macrophage | CD163 | 0.48 | *** | 0.66 | *** |
|  | VSIG4 | 0.51 | *** | 0.51 | * |
|  | MS4A4A | 0.47 | *** | 0.55 | ** |

STAD, stomach adenocarcinoma. TAM, Tumor-associated macrophage. Tumor, correlation analysis in tumor tissue of TCGA. Normal, correlation analysis in normal tissue of TCGA. *P < 0.01; **P < 0.001; ***P < 0.0001.
